# Healthy ageing for all? Comparisons of socioeconomic inequalities in health expectancies over two decades in the Cognitive Function and Ageing Studies I and II

**DOI:** 10.1093/ije/dyaa271

**Published:** 2021-01-09

**Authors:** Holly Q Bennett, Andrew Kingston, Gemma Spiers, Louise Robinson, Lynne Corner, Clare Bambra, Carol Brayne, Fiona E Matthews, Carol Jagger

**Affiliations:** 1 Population Health Sciences Institute, Faculty of Medical Sciences, Newcastle University, Newcastle, UK; 2 Department of Public Health and Primary Care, University of Cambridge, Cambridge, UK

**Keywords:** Life expectancy, health expectancy, disability, dependency, deprivation, social class

## Abstract

**Background:**

Despite increasing life expectancy (LE), cross-sectional data show widening inequalities in disability-free LE (DFLE) by socioeconomic status (SES) in many countries. We use longitudinal data to better understand trends in DFLE and years independent (IndLE) by SES, and how underlying transitions contribute.

**Methods:**

The Cognitive Function and Ageing Studies (CFAS I and II) are large population-based studies of those aged ≥65 years in three English centres (Newcastle, Nottingham, Cambridgeshire), with baseline around 1991 (CFAS I, *n* = 7635) and 2011 (CFAS II, *n *= 7762) and 2-year follow-up. We defined disability as difficulty in activities of daily living (ADL), dependency by combining ADLs and cognition reflecting care required, and SES by area-level deprivation. Transitions between disability or dependency states and death were estimated from multistate models.

**Results:**

Between 1991 and 2011, gains in DFLE at age 65 were greatest for the most advantaged men and women [men: 4.7 years, 95% confidence interval (95% CI) 3.3–6.2; women: 2.8 years, 95% CI 1.3–4.3]. Gains were due to the most advantaged women having a reduced risk of incident disability [relative risk ratio (RRR):0.7, 95% CI 0.5–0.8], whereas the most advantaged men had a greater likelihood of recovery (RRR: 1.8, 95% CI 1.0–3.2) and reduced disability-free mortality risk (RRR: 0.4, 95% CI 0.3–0.6]. Risk of death from disability decreased for least advantaged men (RRR: 0.7, 95% CI 0.6–0.9); least advantaged women showed little improvement in transitions. IndLE patterns across time were similar.

**Conclusions:**

Prevention should target the most disadvantaged areas, to narrow inequalities, with clear indication from the most advantaged that reduction in poor transitions is achievable.

**Key Messages**

- The most advantaged men and women in 2011 spent a higher proportion of remaining life at age 65 disability free or independent than their counterparts in 1991.
- Gains in disability-free life expectancy in the most advantaged between 1991 and 2011 differed between the genders because the most advantaged men in 2011 had a greater likelihood of recovery from disability, but the most advantaged women in 2011 had a lower likelihood of disability onset.
- More advantaged men and women in 2011 had both an increased likelihood of recovery from dependency, and a lower likelihood of dependency onset, compared with those in 1991.
- Compared with the gains in health expectancies experienced by the most advantaged men and women at age 65, the least advantaged men and women saw little change in the percentage of life spent disability free or independent at age 65, between 1991 and 2011.
- The least advantaged men in 2011 had a lower risk of death from disability/dependency than those in 1991, which resulted in more years spent with disability or dependency; in contrast, the least advantaged women did not experience any positive changes in transitions.

## Introduction

Countries worldwide are experiencing population ageing because of rising life expectancy (LE), but many do not experience healthy ageing. Years lived in good health, or without disability, combining morbidity and mortality through health expectancies (HE), have increased over time in most countries worldwide. A critical question for society however, is whether HE gains exceed those in LE thus compressing morbidity, or whether there is expansion of morbidity through an increase in years with ill health or disability, or a dynamic equilibrium where years with ill health or disability increase overall but reduce in severity. In countries where the gains in LE and HE have been studied, there is a lack of consistency.^1^ A relative compression of disability has been observed in some countries (USA, Sweden, Denmark, Norway, China), whereas others (Spain, Japan, Hong Kong, Singapore) have experienced a relative expansion of disability.^1^ Findings are dependent on the populations studied; for example in France there is evidence of both compression^2^ and dynamic equilibrium,^3^ and in Australia trends differ between men and women.^4^ In the UK expansion of disability and dependency has been evident from specific population studies,^5^^,^^6^ and from national surveys.^7^

Not only do time trends in LE and HE differ between countries, but also within countries, by region or socioeconomic status (SES; e.g. income, education, deprivation). Evidence is emerging that in the UK and the USA, LE is stalling and even declining in the most deprived socioeconomic groups.^8^^,^^9^ Studies at single time points show that SES inequalities in HE exceed those in LE,^10^ with those of lowest SES having the double disadvantage of spending a greater proportion of their shorter lives with ill health and disability.^11^ The difference in HE between the most and least advantaged at age 50 appears similar in England and the USA,^12^ although another study found variations across European cohorts with the smallest difference in Sweden (2.1 years at age 50) and largest in Finland (6.8 years).^13^ Widening or persistent inequalities have been reported across time, the former for Denmark, the USA and Belgium,^14–16^ and the latter for Denmark, France and Norway.^17–19^ The small number of recent studies suggest inequalities in healthy and disability-free life expectancy (DFLE) have widened over time.^20^^,^^21^

In England, DFLE differences have been reported across housing tenure,^22^ educational groups^23^ and wealth.^12^ Increasing inequality trends by area deprivation have also been reported.^24^ To date in England, HE by SES trends were estimated from repeated cross-sectional studies that cannot establish the changes to transitions beneath these trends, specifically whether higher SES groups have delayed onset of disability and greater recovery of independence, in relation to mortality changes.

The aims of our study are 2-fold. First, we describe trends in DFLE and years of independence by SES based on longitudinal data. Second, we determine how changes in the underlying transitions have influenced these trends, specifically which changes in transitions were different between SES groups. The Cognitive Function and Ageing Studies (CFAS I and CFAS II) are cross-generational longitudinal studies providing a unique opportunity to examine these transitions in people aged 65 years and over across two decades.

## Methods

The current ethics for MRC CFAS (or CFAS I) is from Eastern MREC, reference number 05/MRE05/37, and for the mortality data Wales REC 7, reference number 14/WA/1154. The current REC reference number for CFAS II is 07/MRE05/48 from Cambridge REC 4. For further information on past ethical approvals please visit the CFAS website [www.cfas.ac.uk].

### Data

The Cognitive Function and Ageing Studies (CFAS I and CFAS II) are large population-based longitudinal studies in three centres (Newcastle, Nottingham and Cambridgeshire). Both studies randomly sampled those aged 65 years or above from the Family Health Service Authority lists (General Practice), including those living in the community, care homes, nursing homes and semi-dependent housing, and stratified into 65–74 years and ≥75 years. Baseline interviews took place over 1991–93 for CFAS I and 2008–11 for CFAS II, with follow-up interviews 2 years later. Interviews included a broad range of questions on demographics, cognition, health, lifestyle, service use and social contact. More details of the studies are available at: [www.cfas.ac.uk].^25^^,^^26^

In a subsample (weighted towards the cognitively frail), an informant interview was requested with a friend or family member suggested by the participant. Importantly for those cognitively frail, the informant interview covered the same topics as the participant interview so that information from the participant interview could be directly substituted with information from the informant interview to reduce missing data.^27^

### Measures

Disability and dependency were defined as in previous analyses of health expectancies.^5^^,^^6^ Disability was measured by impairment in basic or instrumental activities of daily living (ADL and IADL) and was split into severe disability, mild to moderate disability and no disability (Box 1). Dependency is described by the lapsed time requiring help with ADL, IADL and cognitive impairment (measured by the Mini-Mental State Examination),^28^ using the approach of Isaacs and Neville,^29^ and classifying individuals into independent, low dependency, medium dependency and high dependency (Box 1). Dependency was defined hierarchically with high dependency first to maximally utilize non-missing items.

We defined SES by area-level deprivation, measured in both studies by the Townsend deprivation index,^30^ which is derived from information on employment, household overcrowding and car ownership. The deprivation index was categorized such that the study-specific weighted percentage in each group was approximately 33.3% (baseline weights described below).

### Statistical analysis

To examine transitions between disability or dependency states and death and associated factors, we used discrete time Markov multistate models. Both progression and recovery of disability and dependency were modelled, with death the absorbing state. Disability and dependency were dichotomized into no disability/any disability and independent/any dependency, respectively.

Life expectancy, and life expectancy with and without disability or dependency, were estimated by Interpolated Markov Chain (IMaCh) software version 0.99r19.^31^ This method models time discretely by multinomial logistic regression models, but approximates the underlying continuous time structure by decomposing the interval between interviews into several shorter 1-month steps. Additionally we calculated the age at which 50% of remaining years were spent with, and 50% without, disability (DFLE50%) and similarly for dependency (IndLE50%). Date of death was obtained routinely from the Office for National Statistics for both CFAS I and CFAS II. For comparability of follow-up between CFAS I and CFAS II, deaths were censored at 2 years after the second wave interview.

To accurately estimate HE, models were stratified by sex and study (CFAS I and CFAS II). However, in order to estimate relative changes to the transitions between CFAS I and CFAS II, models were stratified for sex only and study was included as a covariate. For instance, this would provide a direct comparison of the probability of a man in CFAS II transitioning between states to the probability of a man in CFAS I transitioning between the same states. Finally, to determine whether changes in transitions were occurring differentially across the population, models were stratified by sex and SES group with study as a covariate, directly comparing the probability of transitioning for least advantaged men in CFAS II with least advantaged men in CFAS I.

All analyses were inverse probability weighted to ensure population representativeness and account for the sampling design (over-sampling of those aged 75 years and over), initial non-response and study design, and longitudinal attrition (full detail provided in Supplementary material, available as Supplementary data at *IJE* online).

As some of the ADLs needed to define dependency were missing in one of the CFAS I interviews, we conducted a sensitivity analysis in CFAS II (where all ADLs were present) to find an alternative measure for dependency. We sought replacement items based on the hierarchy of disability^32^ and compared tested measures by the prevalence of any dependency (further detail provided in Supplementary material).

## Results

The 7635 participants at baseline in CFAS I had an average age of 75.6 years and 60.8% were women; by the 2-year follow-up, 5156 individuals were re-interviewed and 76% of the 6816 individuals were still alive. At CFAS II baseline, 7762 individuals participated; 56.1% were women and average age was 76.4 years; at the 2-year follow-up, 5288 out of those 7119 still alive (74%) were re-interviewed. The baseline distributions of age, SES, place of residence, disability and dependency and the baseline prevalence of disability and dependency by age group and sex in CFAS I and CFAS II are provided in Supplementary Tables S1 and S2, available as Supplementary data at *IJE* online.

### Time trends in disability-free life expectancy and underlying components

**Figure 1:**
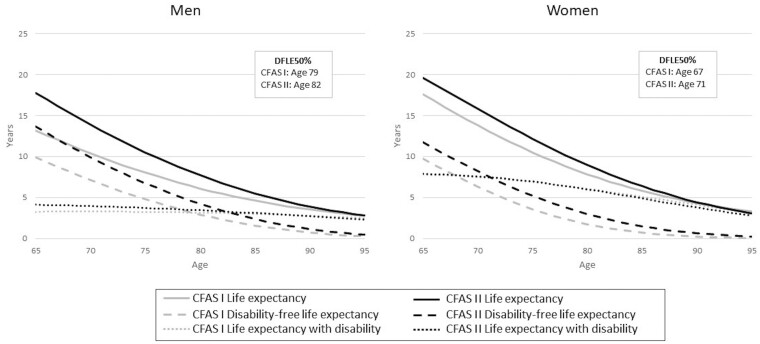
Life expectancy, disability-free life expectancy, life expectancy with disability, and age when years with and without disability are equal (DFLE50%) for men and women in the Cognitive Function and Ageing Studies (CFAS I and CFAS II)

**Figure 2:**
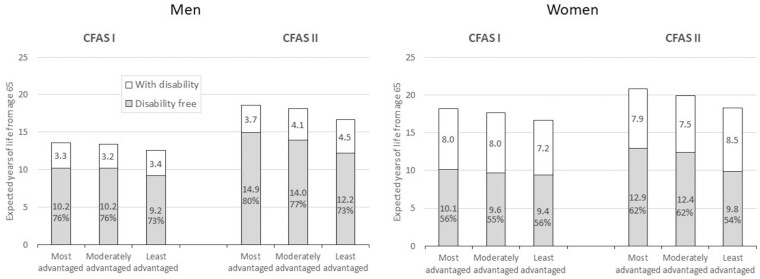
Disability-free life expectancy, life expectancy with disability, and percentage of life expectancy spent disability-free (DFLE%) at age 65 for men and women across socioeconomic status groups in the Cognitive Function and Ageing Studies (CFAS I and CFAS II)

Between CFAS I and CFAS II, men’s LE at age 65 years grew by 4.6 years [95% confidence interval (CI) 3.7 to 5.5 years] with gains in DFLE of 3.7 years (95% CI 2.7-4.8 years), resulting in the proportion of life spent disability-free remaining constant, and a consequent expansion of disability (Table 1). The small increase in life expectancy with disability (DLE) of 0.8 years (95% CI 0.3-1.4 years) was limited to younger ages (Figure 1). When viewed by SES, the gain in DFLE was greatest in the most advantaged and the gain in DLE was greatest in the least advantaged (Figure 2; and Supplementary Table S3, available as Supplementary data at *IJE* online). The proportion of remaining life spent disability-free increased slightly for the most advantaged, who thus experienced a relative compression of disability, but remained stable for the other two groups (Figure 2; and Supplementary Table S3).

**Table 1:** Life expectancy (LE), disability-free life expectancy and life expectancy with disability at age 65 years from the Cognitive Function and Ageing Studies (CFAS I and CFAS II) and the difference between the two studies

	CFAS I	CFAS II	**Difference** **(CFAS II – CFAS I)**
**Men**	**Estimate (95% CI)**	**Estimate (95% CI)**	**Estimate (95% CI)**
Life expectancy (years)	13.2 (12.6–13.8)	17.8 (17.1–18.4)	4.6 (3.7–5.5)
Disability-free LE (years)	9.9 (9.2–10.6)	13.7 (12.9–14.4)	3.7 (2.7–4.8)
Disability-free LE (% of LE)	75.2 (73.5–76.9)	76.8 (75.3–78.4)	1.6 (-0.6–3.9)
LE with disability (years)	3.3 (2.9–3.6)	4.1 (3.7–4.5)	0.8 (0.3–1.4)
LE with disability (% of LE)	24.8 (23.1–26.5)	23.2 (21.6–24.7)	−1.6 (-3.9–0.6)
**Women**	**Estimate (95% CI)**	**Estimate (95% CI)**	**Estimate (95% CI)**
Life expectancy (years)	17.6 (17.0–18.2)	19.6 (18.9–20.3)	2.1 (1.1–3.0)
Disability-free LE (years)	9.8 (9.2–10.4)	11.7 (11.0–12.5)	2.0 (1.0–2.9)
Disability-free LE (% of LE)	55.6 (54.0–57.2)	59.7 (58.0–61.4)	4.1 (1.8–6.5)
LE with disability (years)	7.8 (7.3–8.3)	7.9 (7.3–8.5)	0.1 (-0.7–0.9)
LE with disability (% of LE)	44.4 (42.8–46.0)	40.3 (38.6–42.0)	−4.1 (-6.5 – -1.8)

Models stratified by sex and study. For CFAS I, men total *N* = 2615, CFAS II men *N* = 2866, CFAS I women *N* = 3693 and CFAS II women *N = *3231.

Women’s LE and DFLE at age 65 increased by less than men’s (LE: 2.1 years, 95% CI 1.1-3.0 years; DFLE: 2.0 years, 95% CI 1.0-2.9 years), but with an increase in the proportion of life disability-free of 4.1%age points, and a relative compression of disability (Table 1). Only the most advantaged women experienced this relative compression due to increases in the proportion of life spent disability-free, but their proportion of life disability-free was still lower than that of the least advantaged men who, in contrast, saw little increase in DFLE (Figure 2; and Supplementary Table S3).

**Figure 3:**
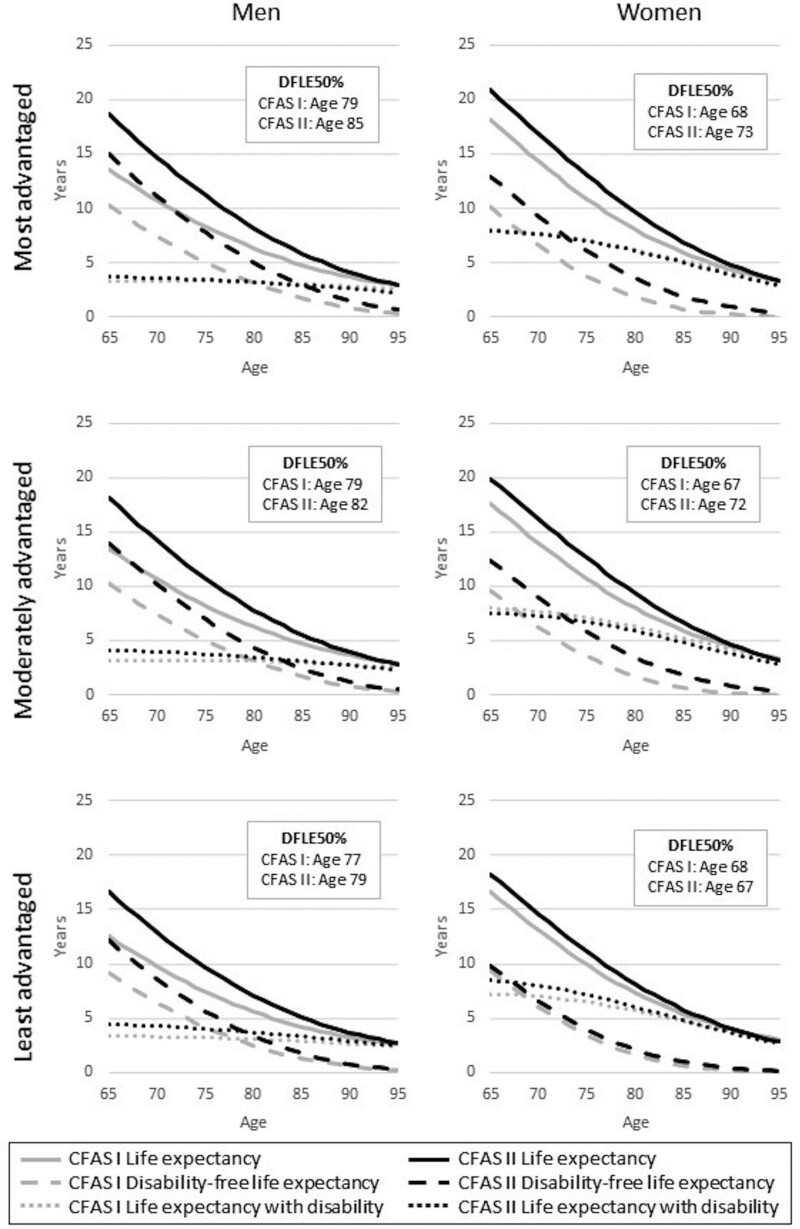
Life expectancy, disability-free life expectancy, life expectancy with disability, and age when years with and without disability are equal (DFLE50%) for men and women in the Cognitive Function and Ageing Studies (CFAS I and CFAS II), by socioeconomic status group

One summary of DFLE and DLE over all ages is the age at which 50% of expected remaining life is disability-free and 50% with disability (DFLE50%). For men overall, DFLE50% increased by 3 years, from 79 years in CFAS I to 82 years in CFAS II (Figure 1), but the gain was greatest in the most advantaged group (6 years) compared with the least advantaged (2 years) (Figure 3). Women’s DFLE50% increased by 3 years, from 68 to 71 years (Figure 1), but the least advantaged experienced a reduction of 1 year, from 68 years to 67 years (Figure 3).

To explore these changes further, we examined differences in the underlying transitions to, and from, disability, and to death, between men and women, over time, and between SES groups. For men, the overall expansion of disability was a result of lower risks of death (50% from no disability and 20% from disability) in CFAS II compared with CFAS I, despite a 20% decrease in risk of incident disability (Table 2). Between CFAS I and CFAS II, more advantaged men experienced a reduction in the risk of incident disability (30% reduction), an increase in the likelihood of recovery to a non-disabled state (80% increase), and a substantially reduced risk of death from no disability (60% reduction). More advantaged women experienced a reduction in the risk of incident disability of the same level as men. Together these provide a huge improvement in life experience for the most advantaged. In contrast, the least advantaged men experienced a reduction in death from disability (30% reduction) between the two studies i.e. prolongation of life with disability, whereas there was little evidence of improvement in transitions for the least advantaged women (Table 2).

**Table 2:** Relative risk ratio (RRR) of transitioning between disability states in the second Cognitive Function and Ageing Study (CFAS II) compared with CFAS I, overall and by deprivation group, 95% confidence interval (CI) in parentheses

		RRR (95% CI)			
Gender	Socioeconomic status	No disability to disability	No disability to death	Disability to no disability	Disability to death
Men	All	0.8 (0.6–0.9)	0.5 (0.4–0.6)	1.2 (0.8–1.6)	0.8 (0.7–0.9)
	Most advantaged	0.9 (0.6–1.2)	0.4 (0.3–0.6)	1.8 (1.0–3.2)	1.0 (0.8–1.2)
	Mid advantaged	0.7 (0.5–1.0)	0.4 (0.2–0.7)	1.0 (0.6–1.8)	0.8 (0.7–1.0)
	Least advantaged	0.7 (0.5–1.0)	0.7 (0.4–1.3)	1.0 (0.5–1.8)	0.7 (0.6–0.9)
Women	All	0.7 (0.6–0.8)	0.7 (0.4–1.1)	1.1 (0.9–1.4)	0.9 (0.8–1.0)
	Most advantaged	0.7 (0.5–0.8)	0.7 (0.4–1.6)	0.8 (0.6–1.2)	0.9 (0.8–1.1)
	Mid advantaged	0.6 (0.5–0.8)	0.7 (0.4–1.4)	1.5 (1.0–2.4)	0.9 (0.8–1.1)
	Least advantaged	0.9 (0.7–1.2)	0.5 (0.2–1.6)	1.1 (0.7–1.6)	0.9 (0.7–1.0)

For overall RRR, models stratified by sex with study as covariate. For socioeconomic status (SES) group RRR, models stratified by sex and SES group with study as covariate.

### Time trends in independent life expectancy and underlying components

Similar patterns to DFLE were evident for independent life expectancy (IndLE) at age 65. Both IndLE and dependent life expectancy (DepLE) increased for men, but only IndLE increased for women, the proportion of remaining life spent independent being similar in CFAS I and CFAS II for men (expansion of dependency) but increasing for women (compression of dependency) (Supplementary Table S4, available as Supplementary data at *IJE* online). The most advantaged men experienced a gain in the proportion of years spent independent and therefore a relative compression of dependency, as did the two most advantaged groups of women. The least advantaged men saw an increase in the proportion of life with dependency and thus an expansion of dependency (Supplementary Figure S1 and Supplementary Table S5, available as Supplementary data at *IJE* online).

**Figure 4:**
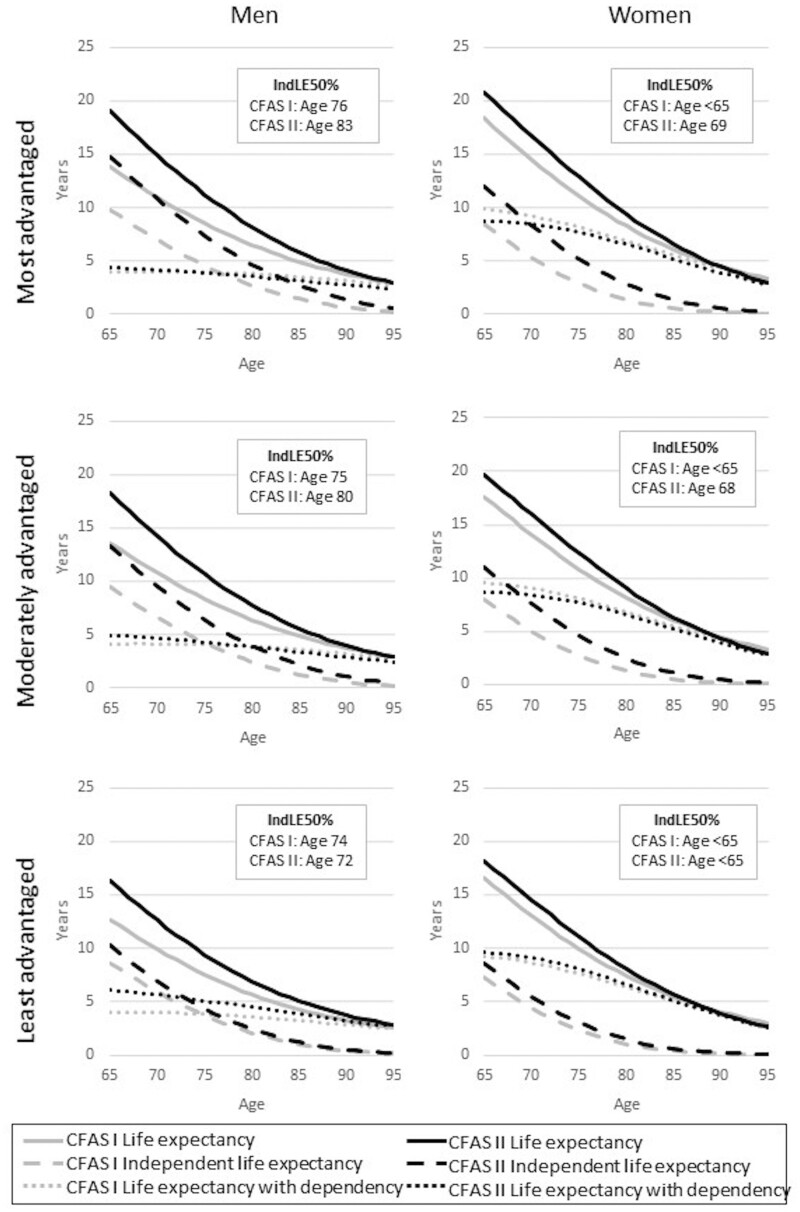
Life expectancy, independent life expectancy, life expectancy with dependency, and age when years with and without dependency are equal (IndLE50%) for men and women in the Cognitive Function Ageing Studies (CFAS I and CFAS II), by socioeconomic status group

The age at which the expected remaining years independent or dependent are equal (IndLE50%) increased between CFAS I and CFAS II: for men (from 75 to 79 years) and for women (from below 65 years to 67 years) (Supplementary Figure S2, available as Supplementary data at *IJE* online). Gains in IndLE50% were greatest for the most advantaged men and women, whereas the least advantaged men experienced 2 years’ reduction (Figure 4).

Findings for IndLE and DepLE were again a result of lower risks in CFAS II (compared with CFAS I) of becoming dependent for both men and women overall (particularly for the more advantaged men) and lower risks of death for men (Supplementary Table S6, available as Supplementary data at *IJE* online).

## Discussion

Widening socioeconomic inequalities in health are evident in a number of countries ^12^^,^^13^^,^^20^^,^^33^ from repeated cross-sectional data. However, little is known about the influences on how people from different periods move between health and independence to disability and death. In this cross-generational population-based study of people aged 65 years and over, with 2-year follow-up at each stage, we report, for the first time, the relative contribution of the underlying transitions to trends in DFLE and IndLE at older ages. In England, comparative trends over 1991–2011 in LE and DFLE at age 65 indicated expansion of disability for men and a relative compression of disability for women overall. This resulted from reductions in the risk of death for men, and in incident disability for men and women over the period. Although overall men experienced an expansion of disability, the most advantaged experienced a relative compression of disability, a pattern also found in the most advantaged women only. The least advantaged men and women had the smallest gains in LE and DFLE and the largest gains in years with disability, resulting from little reduction in incident disability over the period.

The age at which the expected years with and without disability cross (DFLE50%) further illustrated the unequal way in which health and disability have changed in the context of reduced mortality over two decades, particularly for the least advantaged older women. In 1991 DFLE50% was the same, 68 years, for the most and least advantaged women. By 2011 DFLE50% for the most advantaged women had risen to 73 years, whilst falling to 67 years for the least advantaged. Nevertheless, DFLE50% for the most advantaged women in 2011 was 6 years less than that for the least advantaged men. Overall, and by socioeconomic group, IndLE were very similar, although, for the least advantaged men, the age at which years with and without dependency were equal fell by 2 years.

### Strengths and limitations

Both CFAS I and CFAS II are large population-representative studies conducted two decades apart, and with the same sampling strategy that provides robust comparisons across time. The baseline samples included individuals living at home, in care settings and in semi-dependent housing, thus providing results for the total population. This is particularly important for robust estimation of time trends as the proportion of older people entering care homes, and their health profile, has changed considerably over time.^34^ Moreover, we included informant interviews to minimize missing data for the cognitively frail. Our measure of SES, area-level deprivation, is both a strength and a limitation. As a measure of SES in late life, it is more representative of an individual’s current circumstances than occupational status which may have ceased many years previously, or is problematic for these cohorts of women. However it is not an individual measure, such as wealth or pension income, which were not measured in CFAS, rather being reflective of the neighbourhood. There were other limitations to this study that concern the populations studied, response rates, measures used and analytical approaches. First, ethnic minorities are under-represented in CFAS. This under-representation will have increased over time with the ageing of those populations, but this might affect socioeconomic groups differentially. The initial response was lower in CFAS II than in CFAS I, though factors associated with non-response remained stable^35^ and were accounted for in the inverse probability weighting. In terms of measures used, information on two ADLs was not present for one of the CFAS I waves. We conducted a sensitivity analysis in CFAS II, where all ADLs were present, and found an alternative measure having high agreement with the original dependency measure (Supplementary Table S7, available as Supplementary data at *IJE* online). Finally, there were some limitations concerning the analytical approach. Although CFAS I had follow-up interviews at 2, 6 (Cambridgeshire only) and 10 years, we had to limit analysis to match the 2-year follow-up only in CFAS II, thereby restricting the number of transitions observed. Last, given how few individuals recover from severe disability to no disability or from high dependency back to independence, severity could not be modelled as all transitions need to be present for IMaCh. The association between deprivation and transitions between mild/moderate and severe disability or low, moderate and high dependency may therefore differ.

### Interpretation

Social inequalities in the transitions to disability evident in our study were also found in the Whitehall II study, with socioeconomic status shaping the onset of multimorbidity, frailty and disability, but not the risk of mortality after onset.^36^ However, our analysis of men and women separately found the least advantaged men having a reduced risk of death only where disability was already present, thus extending years with disability and being most likely associated with tertiary prevention. Primary prevention strategies focusing on proximal factors such as diet, obesity, smoking, and hypertension have appeared to benefit only the most advantaged, possibly because the least advantaged may be less likely to adapt behaviours if they perceive their life expectancy is limited.^37^

Additionally, there is a role for action outside the health and care system and traditional prevention approaches focused on lifestyle.^38^ Evidence reviews from the World Health Organization^39^ and national and international governments^40^ advocate action to tackle the social determinants of health (e.g. housing and employment conditions, income) as a way of reducing health inequalities and improving healthy life expectancy for all. Comparative analysis of changes in self-rated health between 1991 and 2010, the same period as our study, and a period of recession in England and Sweden, confirmed the deterioration in perceived health in the least advantaged women and improved health in the most advantaged in England.^41^ However, in Sweden health improved across all socioeconomic groups in the context of a more universally supportive welfare system in times of recession. Comparisons of SES differences in health expectancies from longitudinal data across four European cohorts also found the smallest inequalities in Sweden.^13^ The ’10 years on’ report from the 2010 Marmot review documents the differential effect of the large funding cuts on deprived areas which have undermined their capacity to improve the social determinants of health.^42^ The reduction of the health inequalities weighting within the NHS budget, from 15% to 10%, in 2015 will have contributed to this.

Our detailed analysis provides initial insight into why healthy and independent life expectancies across generations have changed differentially according to area deprivation. They have direct relevance for the government target of increasing healthy life expectancy by 5 years while narrowing the gap between the richest and poorest.^43^ Our study suggests that meeting this target will require considerable efforts across all the types of prevention that we use in society: upstream (primary), early detection and screening (secondary) and mitigation (tertiary). Action needs to be taken on improving individual health behaviours, but also encompassing wider determinants (access to care, improved job opportunities, community initiatives), as well as better understanding the barriers from those most affected. Moreover, in the light of suggestions that mortality from COVID-19 is higher in men and in more deprived communities,^44^ our findings of the prolongation of years spent with disability and dependency in the least advantaged men may provide some explanation.

CFAS I and CFAS II data are available upon request after approval from the CFAS management committee. More information and application forms can be accessed at [http://www.cfas.ac.uk/cfas-i/data/#cfasi-data-request] for CFAS I and [http://www.cfas.ac.uk/cfas-ii/cfasii-data] for CFAS II.

## Supplementary data

Supplementary data are available at *IJE* online.

## Funding

This work was supported by the Dunhill Medical Trust and the National Institute for Health Research (NIHR) Policy Research Programme, conducted through the NIHR Older People and Frailty Policy Research Unit, PR-PRU-1217–21502. The views expressed are those of the authors and not necessarily those of the NIHR or the Department of Health and Social Care. CFAS II was supported by the UK Medical Research Council (MRC; research grant G0601022), Alzheimer’s Society (Grant Ref: 294) and received support from the UK National Institute for Health Research (NIHR) comprehensive clinical research networks in West Anglia and Trent, and the Dementias and Neurodegenerative Disease Research Network in Newcastle. MRC CFAS (including CFAS I areas) was funded by the MRC and the UK National Health Service (NHS). H.B. is supported by the Dunhill Medical Trust (grant number RPGF1806\44), and A.K. by a Newcastle University Research Fellowship. This research was undertaken within the UK NIHR collaboration for leadership in applied health research and care for Cambridgeshire and Peterborough and the Cambridge Biomedical Research Centre infrastructures, Nottingham city and Nottinghamshire county NHS primary care trusts, and UK NIHR Policy Research Programme, conducted through the NIHR Older People and Frailty Policy Research Unit, PR-PRU-1217–21502.

## Supplementary Material

dyaa271_Supplementary_Data
