# Supplementary material for: Healthy ageing for all? Comparisons of socioeconomic inequalities in health expectancies over two decades in the Cognitive Function and Ageing Studies I and II
Source: Int J Epidemiol. 2021 Jan 9;50(3):841–51. doi: 10.1093/ije/dyaa271 (PMC8271204; doi:10.1093/ije/dyaa271)
Supplement: dyaa271_Supplementary_Data [file dyaa271_supplementary_data.docx]

**SUPPLEMENTARY MATERIAL**

*Statistical Methods*

Analyses were inverse probability weighted to ensure population representativeness and account for the sampling design (oversampling of those aged 75 years and over). To account for initial non-response and study design: all individuals (including those who died before the censoring date (two years after the two-year follow up interview) were weighted for age at baseline, sex and socioeconomic status (SES). To account for longitudinal attrition: those still alive at the censoring date were excluded from the analysis if they did not participate in the follow-up interviews, as they would not contribute to the likelihood. Since these participants were more likely to have severe disability, which might underestimate recovery and overestimate mortality from the disabled state, an additional weight was estimated based on age, sex, centre, cognitive function, disability, education, social class, deprivation, number of health conditions, self-rated health, smoking, at baseline interview. We applied this weighting in addition to the baseline sampling weight to participants who had not died by the censoring date.

**Supplementary Table S1**: Numbers at baseline and prevalence in the Cognitive Function and Ageing Studies (CFAS I and CFAS II).

|  |  | **CFAS I** | **CFAS II** |
| --- | --- | --- | --- |
|  |  | **% (n)** | **% (n)** |
| N |  | (7635) | (7762) |
| Age group | 65-69 | 25.0 (1981) | 23.0 (1939) |
|  | 70-74 | 22.8 (1776) | 22.7 (1873) |
|  | 75-79 | 22.5 (1725) | 20.5 (1624) |
|  | 80-84 | 17.7 (1308) | 17.5 (1278) |
|  | 85-89 | 8.5 (615) | 10.5 (737) |
|  | 90+ | 3.5 (230) | 5.8 (311) |
| Gender | Men | 39.2 (3045) | 43.9 (3534) |
|  | Women | 60.8 (4590) | 56.1 (4228) |
| Place of residence | Living in the community | 86.0 (6599) | 89.5 (7083) |
|  | Semi-dependent housing | 9.1 (683) | 7.2 (482) |
|  | Care settings | 4.8 (346) | 3.3 (197) |
| Deprivation | Most advantaged | 33.5 (2561) | 33.2 (2940) |
|  | Mid advantaged | 33.2 (2525) | 33.3 (2659) |
|  | Least advantaged | 33.4 (2549) | 33.5 (2163) |
| Disability | None | 68.5 (5236) | 63.4 (4978) |
|  | Mild/Moderate | 14.1 (1048) | 21.1 (1498) |
|  | Severe | 17.5 (1267) | 15.5 (1002) |
| Dependency | Independent | 60.4 (4574) | 54.5 (4300) |
|  | Low dependency | 28.8 (2117) | 33.4 (2344) |
|  | Medium dependency | 6.8 (488) | 6.1 (410) |
|  | High dependency | 3.9 (281) | 6.0 (402) |

**Supplementary Table S2**: Number of people (n) and prevalence of disability or dependency within in each age and sex group in the Cognitive Function and Ageing Studies (CFAS I and CFAS II).

|  | **Men** | | **Women** | |
| --- | --- | --- | --- | --- |
| **Age** | **CFAS I % (n)** | **CFAS II % (n)** | **CFAS I % (n)** | **CFAS II % (n)** |
| **Disability** |  |  |  |  |
| 65-69 | 11.3 (103) | 15.5 (135) | 18.4 (196) | 19.4 (177) |
| 70-74 | 13.9 (108) | 18.5 (155) | 20.9 (208) | 29.7 (270) |
| 75-79 | 22.5 (154) | 23.6 (165) | 33.5 (340) | 42.8 (348) |
| 80-84 | 36.3 (161) | 33.8 (170) | 53.0 (446) | 59.8 (410) |
| 85-89 | 60.5 (98) | 55.7 (138) | 72.1 (312) | 75.1 (297) |
| 90+ | 71.7 (25) | 75.0 (53) | 88.2 (164) | 91.0 (158) |
| **Dependency** |  |  |  |  |
| 65-69 | 16.2 (146) | 22.0 (197) | 23.6 (250) | 25.3 (229) |
| 70-74 | 18.1 (140) | 28.2 (239) | 30.6 (304) | 38.3 (351) |
| 75-79 | 30.6 (208) | 35.9 (253) | 44.5 (449) | 54.2 (440) |
| 80-84 | 48.5 (212) | 45.6 (232) | 65.0 (536) | 68.9 (483) |
| 85-89 | 68.2 (105) | 67.6 (167) | 82.5 (348) | 83.3 (324) |
| 90+ | 82.3 (28) | 83.8 (61) | 94.0 (160) | 95.5 (180) |

**Supplementary Table S3**: Life expectancy (LE), years spent disability-free and years spent with any disability at age 65 years from the Cognitive Function and Ageing Studies (CFAS I and CFAS II) and the difference between the two studies (CFAS II – CFAS I), by socioeconomic status (SES) group and gender. Models stratified by sex and study with SES covariate. Estimates given with 95% confidence intervals in parentheses (95% CI).

|  | **Men** | | | **Women** | | | |
| --- | --- | --- | --- | --- | --- | --- | --- |
|  | **CFAS I** | **CFAS II** | **Difference** | **CFAS I** | **CFAS II** | **Difference** | |
| **Most advantaged** |  |  |  |  |  |  | |
| Life expectancy (years) | 13.5 (12.7 – 14.4) | 18.6 (17.6 – 19.6) | 5.1 (3.8 – 6.4) | 18.2 (17.4 – 19.0) | 20.8 (19.7 – 21.9) | 2.7 (1.3 – 4.0) | |
| Disability free (years) | 10.2 (9.3 – 11.2) | 14.9 (13.8 – 16.0) | 4.7 (3.3 – 6.2) | 10.1 (9.3 – 10.9) | 12.9 (11.7 – 14.2) | 2.8 (1.3 – 4.3) | |
| Disability free (% of LE) | 75.6 (72.8 – 78.4) | 80.2 (77.9 – 82.5) | 4.6 (0.6 – 8.6) | 55.7 (52.9 – 58.5) | 62.1 (59.4 – 64.8) | 6.4 (2.2 – 10.7) | |
| With disability (years) | 3.3 (2.8 – 3.8) | 3.7 (3.1 – 4.3) | 0.4 (-0.4 – 1.2) | 8.0 (7.4 – 8.7) | 7.9 (6.9 – 8.8) | -0.2 (-1.3 – 1.0) | |
| With disability (% of LE) | 24.4 (21.6 – 27.2) | 19.8 (17.5 – 22.1) | -4.6 (-8.6 – -0.6) | 44.3 (41.5 – 47.1) | 37.9 (35.2 – 40.6) | -6.4 (-10.7 – -2.2) | |
| **Mid advantaged** |  |  |  |  |  |  | |
| Life expectancy (years) | 13.4 (12.5 – 14.4) | 18.1 (17.1 – 19.1) | 4.7 (3.3 – 6.1) | 17.6 (16.6 – 18.6) | 19.9 (18.7 – 21.0) | 2.3 (0.8 – 3.8) | |
| Disability free (years) | 10.2 (9.2 – 11.3) | 14.0 (12.8 – 15.1) | 3.7 (2.2 – 5.3) | 9.6 (8.7 – 10.6) | 12.4 (11.1 – 13.6) | 2.7 (1.2 – 4.3) | |
| Disability free (% of LE) | 76.2 (73.3 – 79.0) | 77.2 (74.5 – 79.8) | 1.0 (-2.9 – 4.9) | 54.8 (52.0 – 57.5) | 62.3 (59.5 – 65.1) | 7.5 (3.5 – 11.5) |  |
| With disability (years) | 3.2 (2.6 – 3.8) | 4.1 (3.5 – 4.8) | 0.9 (0.1 – 1.8) | 8.0 (7.1 – 8.8) | 7.5 (6.6 – 8.4) | -0.5 (-1.7 – 0.8) | |
| With disability (% of LE) | 23.8 (21.0 – 26.7) | 22.8 (20.2 – 25.5) | -1.0 (-4.9 – 2.9) | 45.2 (42.5 – 48.0) | 37.7 (34.9 – 40.5) | -7.5 (-11.5 – -3.5) | |
| **Least advantaged** |  |  |  |  |  |  | |
| Life expectancy (years) | 12.5 (11.5 – 13.5) | 16.6 (15.6 – 17.6) | 4.1 (2.7 – 5.5) | 16.6 (15.2 – 18.1) | 18.3 (17.2 – 19.4) | 1.6 (-0.2 – 3.5) | |
| Disability free (years) | 9.2 (8.1 – 10.3) | 12.2 (11.0 – 13.3) | 3.0 (1.4 – 4.6) | 9.4 (8.3 – 10.4) | 9.8 (8.7 – 11.0) | 0.4 (-1.1 – 2.0) | |
| Disability free (% of LE) | 73.2 (70.1 – 76.4) | 73.1 (70.0 – 76.3) | -0.1 (-4.3 – 4.1) | 56.4 (53.5 – 59.3) | 53.7 (50.3 – 57.0) | -2.8 (-6.9 – 1.4) | |
| With disability (years) | 3.4 (2.8 – 3.9) | 4.5 (3.7 – 5.2) | 1.1 (0.2 – 2.0) | 7.2 (6.3 – 8.2) | 8.5 (7.5 – 9.5) | 1.2 (-0.2 – 2.6) | |
| With disability (% of LE) | 26.8 (23.6 – 29.9) | 26.9 (23.7 – 30.0) | 0.1 (-4.1 – 4.3) | 43.6 (40.7 – 46.5) | 46.3 (43.0 – 49.7) | 2.8 (-1.4 – 6.9) | |

**Supplementary Table S4**: Life expectancy (LE), years spent independent and years spent with any dependency at age 65 years from the Cognitive Function and Ageing Studies (CFAS I and CFAS II) and the difference between the two studies. Models stratified by sex and study. For CFAS I men total N=2615, CFAS II men N=2866, CFAS I women N=3693 and CFAS II women N=3231.

|  | **CFAS I** | **CFAS II** | **Difference**  **(CFAS II – CFAS I)** | |
| --- | --- | --- | --- | --- |
| **Men** | **Estimate (95% CI)** | **Estimate (95% CI)** | **Estimate (95% CI)** | |
| Life expectancy (years) | 13.4 (12.7 – 14.0) | 17.9 (17.3 – 18.5) | 4.5 (3.6 – 5.4) | |
| Independent (years) | 9.3 (8.6 – 10.1) | 12.8 (12.0 – 13.6) | 3.5 (2.4 – 4.5) | |
| Independent (% of LE) | 69.8 (68.0 – 71.5) | 71.5 (69.8 – 73.1) | 1.7 (-0.7 – 4.1) | |
| With dependency (years) | 4.0 (3.6 – 4.5) | 5.1 (4.7 – 5.6) | 1.1 (0.4 – 1.7) | |
| With dependency (% of LE) | 30.2 (28.5 – 32.0) | 28.5 (26.9 – 30.2) | -1.7 (-4.1 – 0.7) | |
| **Women** | **Estimate (95% CI)** | **Estimate (95% CI)** | **Estimate (95% CI)** | |
| Life expectancy (years) | 17.6 (17.0 – 18.3) | 19.5 (18.8 – 20.2) | 1.9 (0.9 – 2.8) | |
| Independent (years) | 8.0 (7.4 – 8.7) | 10.5 (9.8 – 11.3) | 2.5 (1.5 – 3.5) | |
| Independent (% of LE) | 45.4 (43.8 – 47.0) | 54.0 (52.3 – 55.7) | 8.6 (6.2 – 11.0) |  |
| With dependency (years) | 9.6 (9.0 – 10.3) | 9.0 (8.3 – 9.6) | -0.7 (-1.6 – 0.3) | |
| With dependency (% of LE) | 54.6 (53.0 – 56.2) | 46.0 (44.3 – 47.7) | -8.6 (-11.0 – -6.2) | |

**Supplementary Table S5**: Life expectancy (LE), years spent independent and years spent with any dependency at age 65 years from the Cognitive Function and Ageing Studies (CFAS I and CFAS II) and the difference between the two studies, by socioeconomic status (SES) group and gender. Models stratified by sex and study with SES covariate.

|  | **Men** | | | **Women** | | |
| --- | --- | --- | --- | --- | --- | --- |
|  | **CFAS I** | **CFAS II** | **Difference** | **CFAS I** | **CFAS II** | **Difference** |
| **Most advantaged** |  |  |  |  |  |  |
| Life expectancy (years) | 13.8 (13.0 – 14.6) | 19.1 (18.1 – 20.0) | 5.3 (4.0 – 6.5) | 18.3 (17.6 – 19.1) | 20.7 (19.7 – 21.8) | 2.4 (1.0 – 3.7) |
| Independent (years) | 9.8 (8.9 – 10.7) | 14.7 (13.6 – 15.9) | 4.9 (3.5 – 6.4) | 8.5 (7.7 – 9.3) | 12.0 (10.8 – 13.2) | 3.5 (2.1 – 5.0) |
| Independent (% of LE) | 71.0 (68.1 – 73.9) | 77.2 (74.8 – 79.6) | 6.2 (2.0 – 10.4) | 46.2 (43.4 – 49.1) | 57.9 (55.1 – 60.6) | 11.6 (7.3 – 15.9) |
| With dependency (years) | 4.0 (3.5 – 4.5) | 4.3 (3.6 – 5.1) | 0.3 (-0.5 – 1.2) | 9.9 (9.1 – 10.6) | 8.7 (7.7 – 9.7) | -1.1 (-2.4 – 0.1) |
| With dependency (% of LE) | 29.0 (26.1 – 31.9) | 22.8 (20.4 – 25.2) | -6.2 (-10.4 – -2.0) | 53.8 (50.9 – 56.6) | 42.1 (39.4 – 44.9) | -11.6 (-15.9 – -7.3) |
| **Mid advantaged** |  |  |  |  |  |  |
| Life expectancy (years) | 13.6 (12.6 – 14.5) | 18.2 (17.2 – 19.2) | 4.6 (3.3 – 6.0) | 17.6 (16.6 – 18.6) | 19.6 (18.5 – 20.7) | 2.0 (0.5 – 3.5) |
| Independent (years) | 9.4 (8.4 – 10.4) | 13.3 (12.0 – 14.5) | 3.9 (2.3 – 5.5) | 8.0 (7.0 – 9.1) | 11.0 (9.8 – 12.1) | 2.9 (1.4 – 4.5) |
| Independent (% of LE) | 69.4 (66.3 – 72.5) | 73.0 (70.2 – 75.8) | 3.5 (-0.6 – 7.7) | 45.6 (42.8 – 48.4) | 56.0 (53.1 – 58.8) | 10.3 (6.3 – 14.4) |
| With dependency (years) | 4.1 (3.5 – 4.8) | 4.9 (4.2 – 5.7) | 0.8 (-0.2 – 1.8) | 9.6 (8.6 – 10.6) | 8.6 (7.6 – 9.7) | -1.2 (-2.5 – 0.05) |
| With dependency (% of LE) | 30.6 (27.5 – 33.7) | 27.0 (24.2 – 29.8) | -3.5 (-7.7 – 0.6) | 54.4 (51.6 – 57.2) | 44.0 (41.2 – 46.9) | -10.3 (-14.4 – -6.3) |
| **Least advantaged** |  |  |  |  |  |  |
| Life expectancy (years) | 12.7 (11.7 – 13.7) | 16.4 (15.3 – 17.4) | 3.6 (2.2 – 5.0) | 16.6 (15.6 – 17.6) | 18.2 (17.0 – 19.3) | 1.6 (0.1 – 3.1) |
| Independent (years) | 8.7 (7.6 – 9.7) | 10.3 (8.9 – 11.6) | 1.6 (-0.1 – 3.3) | 7.3 (6.4 – 8.3) | 8.5 (7.3 – 9.7) | 1.2 (-0.3 – 2.8) |
| Independent (% of LE) | 68.0 (64.7 – 71.4) | 62.7 (59.2 – 66.1) | -5.3 (-9.8 – -0.9) | 44.1 (41.1 – 47.0) | 47.0 (43.6 – 50.3) | 2.9 (-1.2 – 7.1) |
| With dependency (years) | 4.1 (3.4 – 4.7) | 6.1 (5.3 – 6.9) | 2.0 (1.0 – 3.1) | 9.3 (8.3 – 10.2) | 9.6 (8.5 – 10.8) | -0.2 (-1.6 – 1.2) |
| With dependency (% of LE) | 32.0 (28.6 – 35.3) | 37.3 (33.9 – 40.8) | 5.3 (0.9 – 9.8) | 55.9 (53.0 – 58.9) | 53.0 (49.7 – 56.3) | -2.9 (-7.1 – 1.2) |

**Supplementary Table S6:** Relative Risk Ratio (RRR) of transitioning between dependency states in the second Cognitive Function and Ageing Study (CFAS II) compared to CFAS I, overall and by socioeconomic status (SES) group, 95% confidence interval (CI) in parentheses. Overall RRR models stratified by sex with study covariate. SES group RRR models stratified by sex and SES group with study covariate.

| **Gender** | **Deprivation** | **Independent to dependent** | **Independent to death** | **Dependent to independent** | **Dependent to death** |
| --- | --- | --- | --- | --- | --- |
| Men | ALL | 0.8 (0.7 – 1.0) | 0.4 (0.3 – 0.5) | 1.2 (0.9 – 1.7) | 0.8 (0.7 – 0.9) |
|  | Most advantaged | 0.9 (0.6 – 1.2) | 0.4 (0.2 – 0.6) | 1.6 (1.0 – 2.6) | 1.0 (0.8 – 1.2) |
|  | Mid advantaged | 0.7 (0.5 – 1.0) | 0.4 (0.2 – 0.7) | 1.3 (0.8 – 2.1) | 0.9 (0.7 – 1.1) |
|  | Least advantaged | 0.9 (0.7 – 1.2) | 0.4 (0.2 – 1.0) | 1.0 (0.6 – 1.8) | 0.7 (0.6 – 0.9) |
| Women | ALL | 0.7 (0.6 – 0.8) | 0.8 (0.5 – 1.4) | 1.2 (0.9 – 1.5) | 0.9 (0.9 – 1.0) |
|  | Most advantaged | 0.6 (0.5 – 0.8) | 0.9 (0.4 – 2.1) | 1.0 (0.7 – 1.4) | 1.0 (0.8 – 1.1) |
|  | Mid advantaged | 0.7 (0.6 – 0.9) | 0.8 (0.4 – 1.5) | 1.8 (1.2 – 2.8) | 0.9 (0.8 – 1.1) |
|  | Least advantaged | 0.7 (0.6 – 1.0) | 0.8 (0.3 – 2.4) | 0.9 (0.6 – 1.5) | 0.9 (0.8 – 1.1) |

*RRR from gender separate, study stratified model rather than study and gender separate

**Supplementary Figure S1**: Independent life expectancy, life expectancy with dependence and percentage of life expectancy spent independent at age 65 in CFAS I and CFAS II for men and women, by socioeconomic group.


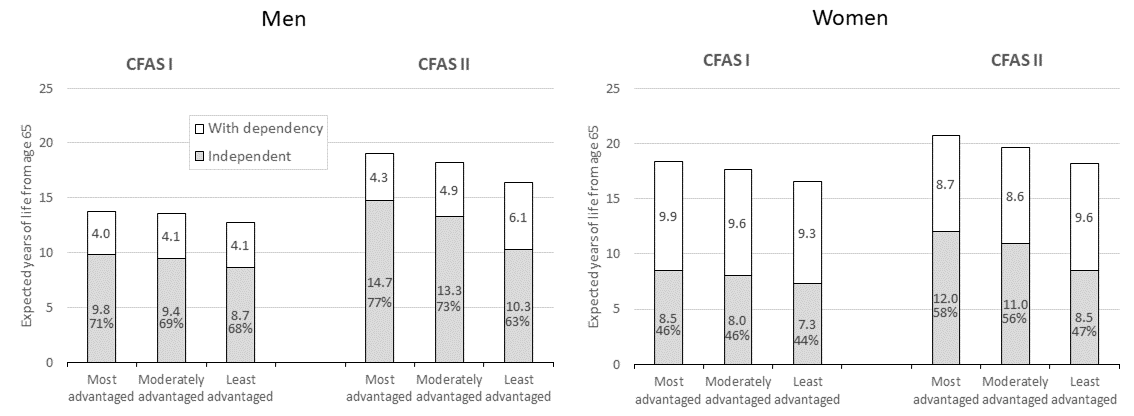


**Supplementary Figure S2**: Life expectancy, years independent, years with any dependency and age where years with and without dependency is equal (IndLE50%) for men and women in the Cognitive Function and Ageing Studies (CFAS I and CFAS II).


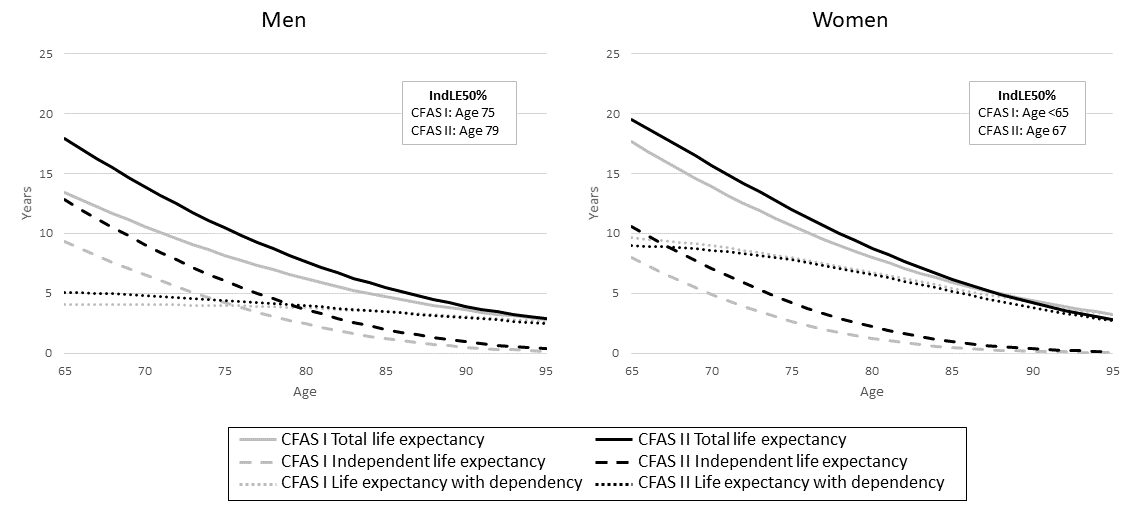


*Sensitivity analysis*

In the first Cognitive Function and Ageing Study (CFAS I) two questions required to define dependency were omitted in one of the interviews (wave c2 – see CFAS website for CFAS I wave structures ^1^) but were present in all other CFAS I and CFAS II interviews. The omitted questions were ‘difficulty with toileting’ to define high dependency and ‘difficulty with light housework’ to define low dependency. Incontinence, difficulty putting on shoes and socks and difficulty with household tasks such as making a cup of tea were tested in variations to replace the omitted questions. The sensitivity analysis was carried out in CFAS II where all the questions were present. Without the omitted variables prevalence of high dependency was low (Alternate 1, Supplementary Table S7) in comparison to the original measure (Supplementary Table S1 and Supplementary Table S7). Initially as a replacement for toileting, difficulty with putting on shoes and socks in addition to incontinence addressed the physical part of getting to the toilet, though the prevalence of high dependency remained low in comparison to the original (Alternate 2, Supplementary Table S7). Then incontinence alone was used as a replacement (Alternate 3, Supplementary Table S7). Difficulty with household tasks such as making a cup of tea was tested as a replacement for difficulty with light housework but in comparison to the original this slightly underestimated independence (Alternate 4, Supplementary Table S7). Given that all levels of dependency would be grouped together to give any dependency Alternate 3 was chosen as a compromise as this would give the most similar prevalence of independence versus any dependency compared with the other alternatives.

**Supplementary Table S7**: Prevalence of dependency in the second Cognitive Function and Ageing Study (CFAS II) at baseline using different replacement variables for missing CFAS I wave c2 variables

|  | **Original^1^** | **Alternate 1^2^** | **Alternate 2^3^** | **Alternate 3^4^** | **Alternate 4^5^** |
| --- | --- | --- | --- | --- | --- |
| Independent | 54.5 | 56.3 | 56.2 | 55.1 | 53.2 |
| Low dependency | 33.4 | 34.1 | 34.1 | 32.2 | 34.1 |
| Medium dependency | 6.1 | 6.5 | 6.3 | 5.9 | 5.9 |
| High dependency | 6.0 | 3.2 | 3.5 | 6.8 | 6.8 |

^1^Original: Including the variables that were omitted from the first Cognitive Function and Ageing Study (CFAS I) wave c2 (same as prevalence given in Supplementary Table S1). ^2^Alternate 1: Excluding variables omitted from CFAS I wave c2 (toileting from high dependency and light housework from low dependency). ^3^Alternate 2: Excluding variables omitted from CFAS I wave c2 and replacing toileting with being incontinent and also having difficulty putting on shoes and socks. ^4^Alternate 3: Excluding variables omitted from CFAS I wave c2 and replacing toileting with being incontinent. ^5^Alternate 4: Excluding variables omitted from CFAS I wave c2, replacing toileting with being incontinent and replacing light housework with difficulty with household tasks such as making a cup of tea.

**References for Supplementary material**

1. Cognitive Function and Ageing Studies. *MRC CFAS Study Design*. 2018 [cited 2019 18/10/2019]; Available from: <http://www.cfas.ac.uk/cfas-i/cfasistudy-design/>
